# ODISSEI Secure Supercomputer - a breakthrough in complex data linkage and applications.

**DOI:** 10.23889/ijpds.v9i5.2752

**Published:** 2024-09-18

**Authors:** Kasia Karpinska

**Affiliations:** 1 Erasmus University Rotterdam / ODISSEI

## Objectives

Antimicrobial resistance (AMR) has become one of the most serious global public health threats. Concerns about AMR resulted in increased monitoring and evaluation of antibiotic prescribing, with primary care responsible for over 80% of these prescriptions. Through this study, we aim to analyse temporal trends and variations in antibiotic prescribing at GP practice level and investigate the association between antibiotic prescribing and demographic, clinical, geographic, and socio-economic characteristics in Northern Ireland.

## Method

The descriptive analyses are complemented by performing the multilevel modelling analysis to identify practice-level determinants of prescribing.

## Results

Changes in standardised antibiotic prescribing rates are evaluated using a linear mixed-effects model with a random intercept for each GP practice. We discuss the changes in antibiotic prescribing rates over time, between-GP practice variations in prescribing, differences in prescribing between urban and urban practices, and the impact of demographic and socio-economic factors on antibiotic prescribing rates.

## Conclusions and Implications

This study contributes to the debate on determinants of antibiotic prescribing in primary care and provides actionable insights to policy makers responsible for antimicrobial stewardship and public health campaigns.

